# A Neuroendocrine Mechanism of Co-Morbidity of Depression-Like Behavior and Myocardial Injury in Rats

**DOI:** 10.1371/journal.pone.0088427

**Published:** 2014-02-13

**Authors:** Wang Xinxing, Liu Wei, Wu Lei, Zhan Rui, Jin Baoying, Qian Lingjia

**Affiliations:** 1 Beijing Institute of Basic Medical Sciences, Beijing, China; 2 Institute of Health & Environmental Medicine, Tianjin, China; 3 Tianjin Occupational Disease Prevention Hospital (Hospital Workers), Tianjin, China; University of Regensburg, Germany

## Abstract

Depression is generally a recurrent psychiatric disorder. Evidence shows that depression and cardiovascular diseases are common comorbid conditions, but the specific pathological mechanisms remain unclear. The purpose of this study is to determine the effects of depression induced by chronic unpredictable mild stress (CUMS) on myocardial injury and to further elucidate the biological mechanism of depression. Rats were used as a model. The CUMS procedure lasted for a total of 8 weeks. After 4 weeks of CUMS, treated rats exhibited a reduced sucrose preference and changes in scores on an open field test, body weight and content of 5-HT in the brain as compared with the values of these variables in controls. These changes indicated depression-like changes in CUMS rats and demonstrated the feasibility of the depression model. In addition, pathological changes in the myocardium and increased cardiomyocyte apoptosis demonstrated that myocardial injury had occurred after 6 weeks of CUMS and had increased significantly by the end of 8 weeks of CUMS. Plasma serotonin (5-HT), norepinephrine (NE) and epinephrine (E), all depression-related neuroendocrine factors, were measured by HPLC-ECD techniques, and the content of plasma corticosterone (GC) was evaluated by an I^125^–cortisol radioactivity immunoassay in control and CUMS rats. The results indicated that 5-HT had decreased, whereas NE, E and GC had increased in CUMS rats, and these factors might be associated with depression-induced myocardial injury. The effects of 5-HT, NE and GC on the survival rate of cultured cardiomyocytes were determined using an orthogonal design. The results showed that 5-HT was a more important factor affecting cell survival than GC or NE. The results suggested that normal blood levels of 5-HT had a cytoprotective effect. The neuroendocrine disorders characterized by decreased 5-HT combined with increased GC and NE mediated the occurrence of depression-induced myocardial injury.

## Introduction

Cardiovascular disease (CVD) is a major cause of death in developed countries [1]. At the beginning of the 20th century, less than 10% of world mortality was related to CVD [2]. The association between CVD and depression is well established and is suggested to be bidirectional [3]. Depressed mood has been linked to CVD risk factors in patients with and without baseline cardiac disease [4], major depression is an independent risk factor for cardiovascular mortality and morbidity [5], [6]. Biological mechanisms that might link these two conditions together include the hypothalamic–pituitary–adrenal (HPA) axis, changes of arterial elasticity and endothelial function [7], [8]. While the exact mechanisms linking depression and increased cardiovascular risk remain poorly understood.

Although the exact biochemical mechanisms remain largely unknown, it is well established that depression and CVD share a common biochemical background that includes the main hormonal mediators of stress [9]. Stress includes etiological mechanisms for depression and heart injury [10], [11]. In animal models, only special stress protocols are available to induce depression-like symptoms. The chronic mild stress model has been widely accepted [12]–[14]. During the establishment of this model, rats are submitted to a regimen of chronic, mild and nontraumatic stressors such as deprivation of food and water, overnight illumination, cage tilt or a change of cage mate. This model is appropriate for the direct study of the pathogenesis, biological mechanisms and treatments of depression and is focused on the behavioral expressions of anhedonia (a central feature of depression characterized by an impaired responsiveness to pleasurable stimuli), which has been examined using sucrose preference tests [15]–[17].

Grippo [18] and colleagues found that rats exposed to chronic mild stress displayed an elevated heart rate, reduced heart rate variability and elevated sympathetic cardiac tone as well as anhedonia and reduced activity level in a running wheel after 4 weeks of stress. Their findings suggest that rats exposed to chronic mild stress are vulnerable to arrhythmic events that, in turn, may influence such additional detrimental cardiac events as myocardial infarction or death. Thus, in the present study, we investigated the comorbidity of depression and heart injury using rats as a model.

The adverse effects of antidepressants on the cardiovascular system have long been recognized. Although antidepressants are generally effective in decreasing depression, their use in patients with CHD is controversial [19], [20]. The safety of both tricyclic antidepressants (TCAs) and selective serotonin reuptake inhibitors (SSRIs) has been questioned [21], [22]. However, some studies have shown that SSRI medication decreases depression symptoms and may improve CHD prognosis in patients with CHD and depression [21], [23]. It is therefore important to explore proper interventional drugs for both disorders. Sertraline is an SSRI that has been used for depression treatment. Heart toxicity due to sertraline has not been observed in animal or human research. Although antiplatelet and protective endothelium effects of sertraline have been reported [24], the results from three currently available epidemiological studies assessing the risk of MI in patients treated with SSRIs are controversial with regard to a potential beneficial effect of SSRIs on CVD risk in depressed patients. One study confirmed that sertraline caused concentration-dependent dilation in reconstructed arteries [22], [25]. A high dose of sertraline was able to fully reverse the level of vascular constriction caused by all types of vasoconstrictors. Sertraline also increased coronary flow in the isolated rat heart in a dose-dependent fashion.

The aim of this study was to hypothesize the Plasma serotonin (5-HT), corticosterone (GC), norepinephrine (NE) and epinephrine (E), all depression-related neuroendocrine factors is closely related to myocardial injury. The neuroendocrine disorders characterized by changed 5-HT, GC and/or NE is the physiology mechanism of depression-induced myocardial injury. Moreover, the present study attempted to assess the therapeutic effect of sertraline on depression and heart injury *in vivo*. The study provides a theoretical reference for the clinical treatment of the comorbidity of depression and cardiovascular diseases.

## Materials and Methods

### 1 Animals

This study was conducted with the approval of the Animal Care and Use Committee of the Beijing Institute of Basic Medical Sciences, China. The protocol was approved by the Committee on the Ethics of Animal Experiments of the Beijing Institute of Basic Medical Sciences (Permit Number: 2012-D-3096). All surgery was performed under sodium pentobarbital (1%, 1 ml/100 g weight, i.p.) anesthesia, and all efforts were made to minimize suffering.

Male Wistar rats (42 days, about 180 g) were used for the experiment, and 8 rats were treated in each group. Rats were acclimated to the surroundings for 1 week before the start of experiment. The animals were maintained on a 12 h light/dark cycle under a controlled temperature of 25±2°C. Food and water were available for the duration of the experiments unless otherwise noted. All animal handling procedures were performed in strict accordance with the guide for the use and care of laboratory animals.

### 2 Sucrose Preference Tests and Body Weight Measurement

Anhedonia, which is a central feature of depression, was defined as a reduction in sucrose preference. The methods for measuring sucrose preference were similar to those described by Muscat and Willner et al. [26], [27]. Rats were trained for adaptation to the taste of a sucrose solution (1%) prior to the start of the experiment. After removing the food and water from each rat’s cage for a period of 20 h, the rats were exposed to water and 1% sucrose solution in pre-weighed plastic bottles that were placed on the cages. The rats were then allowed to consume the fluids for a period of 1 h. The bottles were then removed and weighed to determine the amount (in grams) of each fluid consumed. The tests were conducted and body weights were measured before commencing the CUMS protocol (baseline) and every 2 weeks thereafter for 8 weeks. Sucrose preference was calculated according to the following formula: % sucrose preference = (sucrose intake/total fluid intake) × 100.

### 3 Protocol for Chronic Unpredictable Mild Stress (CUMS)

CUMS is used to model the negative life events of humans. CUMS produces anhedonia in rats, which demonstrates its effectiveness as an animal model [26], . In our experiment, rats were divided into control and CUMS groups according to their sucrose preference in the baseline test, in order to with the same depressive symptoms. The experimental protocol and CUMS procedure were variations of methods described by Grippo et al. [18], [28]. Briefly, The CUMS rats were caged individually, whereas the control rats were caged groups. The CUMS procedure was carried out for a total of 8 weeks. The stressors for CUMS included continuous overnight illumination, a 40° cage tilt along the vertical axis, paired housing, soiled cages (200 mL water spilled into the bedding of each cage), restraint in a small cage (equipped with breathing holes), exposure to an empty water bottle for 1 h immediately following a period of acute water deprivation and food deprivation. All of the stressors were applied individually and continuously, day and night. Control animals were left undisturbed in their home cages except for handling during regular cage cleaning and measuring body weight.

### 4 Open Field Test

Locomotors activity and exploratory behavior were evaluated using an open field test. The open field apparatus was a plywood box measuring 100 cm × 100 cm. All of the walls were painted black. The floor was divided into 25 equal squares. Rats were placed individually in one corner of the apparatus, and their ambulation (number of squares crossed) and immobility frequency were observed for 3 m. An open field test score was calculated as the sum of ambulation and immobility frequency [29].

### 5 Myocardial Morphological Structure Analysis of Rats

Myocardial damage was observed *post-mortem* after 2, 4, 6 and 8 weeks of CUMS using Nagar Olsen-stained paraffin sections of the myocardium. Each section was stained for 10 min with an alum hematoxylin solution. The section was then washed for 5 min with distilled water and stained for 3 min with basic fuchsin staining solution (0.1 g basic fuchsin stain in 100 mL of distilled water). The sections were then washed for 5 to 10 s with distilled water and for 5 s with acetone wash. The sections were then incubated in a 1% picric acid solution (1 g picric acid in 100 mL of acetone) for 5 to 15 s for quick differentiation and sealed with xylene and neutral gum. Red areas were measured with AxioVision under an optical microscope.

The pathological alteration of the rat cardiomyocytes was analyzed using an electron microscope (Hitachi, Japan) according to Coleman’s method [30]. The ventricular muscles of the rat hearts were dissected and fixed overnight in pre-cooled 3% glutaraldehyde and then in 1% osmium. Ultrastructural changes in the myocardium were observed by electron microscopy after being marked with uranium and lead.

### 6 Pharmacological Treatment

Rats were divided into 4 groups randomly after 4 weeks of exposure to CUMS: a control group given only water to drink, a control group that received sertraline hydrochloride (i.g., 10 mg/kg/day) but was not exposed to CUMS, a CUMS group that was not treated with sertraline and a CUMS group that was treated with sertraline. Sertraline is an SSRI and seems to be a safe and efficacious treatment for depression [31]–[33]. The CUMS procedure was carried out for a total of 8 weeks.

### 7 Terminal Deoxynucleotidyl Transferase dUTP nick-end Labeling Assay

After 2, 4, 6 and 8 weeks of CUMS, a terminal deoxynucleotidyl transferase deoxyuridine triphosphate (dUTP) nick-end labeling (TUNEL) assay was used to determine tissue cardiomyocyte apoptosis as previously described [34], the same tissue samples were used as those mentioned in Section 2.5.

### 8 The Determination of Plasma Hormone Levels

Plasma serotonin (5-HT) was detected as previously described [35], briefly, Aliquots of sample (5 µl) were injected onto a microbore HPLC column (Unijet, 100×1 mm, 5 µM ODS, Bioanalytical Systems, Inc, West Lafayette, IN, USA) that was coupled to an amperometric detector (Model LC-4C, BAS, Inc.). A glassy carbon electrode was set at a potential of +650 mV relative to Ag/AgCl reference. Mobile phase consisted of 180 µM Na2EDTA, 150 mM monochloroacetic acid, 125 mM NaOH, and 318 µM sodium octanesulfonic acid, with 4.5% MeOH and 4.5% CH_3_CN per liter of water (final pH = 3.15). Mobile phase was pumped through the column at 60 µl/min (Model 260 D, Teledyne ISCO, Lincoln, NE, USA). Chromatographic data were acquired on-line and exported to a Millennium software system (Waters Associates, Milford, MA, USA). The concentration of 5-HT in dialysate samples was compared to known standards, and the lower limit of detection was 0.05 pg/5 µL (0.047 nM). Norepinephrine (NE) and epinephrine (E) were also measured by high-performance liquid chromatography (HPLC) with electrochemical detection (ECD) [36]. Plasma corticosterone (GC) level was evaluated by a I125–cortisol radioactivity immunoassay in both control and CUMS rats [37].

### 9 Statistical Analyses

Values are presented as the mean ± SE for the indicated experiments and were analyzed by a mixed-design ANOVA, a one-way ANOVA and Student’s *t*-tests as appropriate. A probability value of *P<*0.05 was considered to be statistically significant.

## Results

### 1 Chronic Unpredictable Mild Stress Induces Comorbidity of Depression-like Behavior and Myocardial Injury in Rats

**Figure 1:**
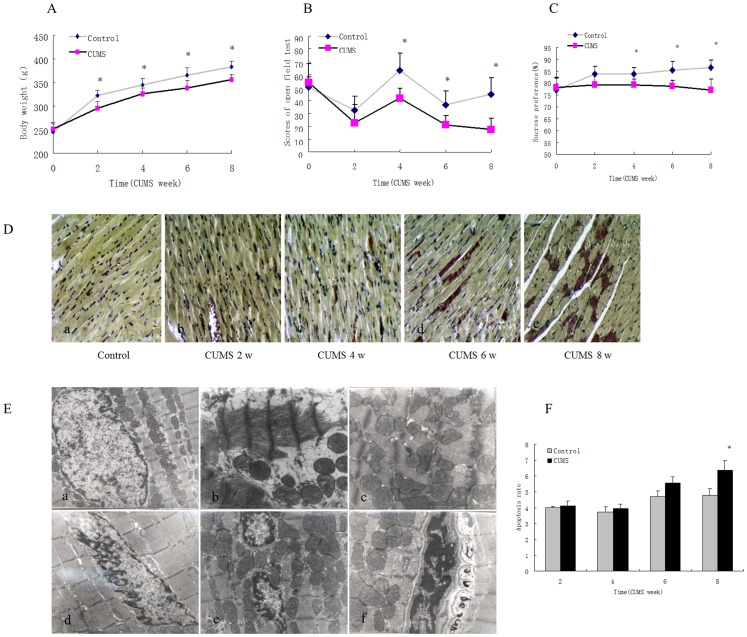
Unpredictable chronic mild stress induces comorbidity of depression-like behavior and myocardial injury in rats. The depression-like behavior and myocardial injury of CUMS rats were measured at 2, 4, 6 and 8 weeks. **A.** The rats were weighed weekly, and the weights are shown. **B.** Locomotor activity and exploratory behavior were evaluated as described in the Materials and Methods section. Values are the mean ± SD from 3 independent experiments, *P<0.05 compared with the control. **C.** A 2-bottle sucrose preference test was used to measure rats’ preferences for sweetened solution over water. Preference was calculated as the percentage of sucrose solution consumed compared to the total fluid intake (sucrose/(sucrose+water) x 100). **D.** Myocardial damage was observed by Nagar Olsen staining. The size and scale of the red-stained range represent the severity of the injury. There was no injury in control group rats (a) or in 2 and 4 week CUMS rats (b, c). Myocardial injury occurred after 6 weeks of CUMS (d) and increased after 8 weeks of CUMS (e). **E.** An electron microscope was used to detect pathological ultrastructural alteration. A myocardial ultrastructure showing normal cardiac nuclear, myofibril structure and mitochondria (×10000) in control group rats is shown (a). Abnormal ultrastructural changes such as myofibril breakage were observed after 8 weeks of CUMS (×10000) (b). Mitochondrial disarrangement, mitochondrial interrupted inner membrane and cristae and mitochondrial vacuoles (×10000) are shown (c, f). Nuclear membrane shrinkage and chromatin margination (×10000) were also seen in 8-week CUMS rats (d, e, f). **F.** Restraint stress increased cardiomyocyte apoptosis as measured by TUNEL. Values represent the group mean ± structural equation modeling (SEM) (n = 8 rats per group). *P<0.05 compared with control, repeated measures ANOVA followed by Tukey’s multiple comparison test.

We found that chronic unpredictable mild stress caused a significant loss of body weight (Fig. 1A) (F = 6.89, t = 2.98, df = 15, P<0.05). The total open field test scores are shown in Figure 1B. The open field test score of CUMS rats decreased significantly compared with the control group after 8 weeks of CUMS (F = 7.42, t = 3.25, df = 15, P<0.05). The sucrose preference test indicates the degree of the animals’ responsiveness to reward. Chronic unpredictable mild stress significantly decreased sucrose preference (Fig. 1C) (F = 7.36, t = 3.12, df = 15, P<0.05). This decrease in sucrose preference is similar to symptoms of depression and anhedonia. Myocardial tissue injury (the myocardial red area in the figures) was observed after 6 and 8 weeks of CUMS (Fig. 1D).

Electron microscopy was used to observe myocardial ultrastructural changes resulting from CUMS to clarify the pathological features of myocardial injury. As shown in Figure 1F, the myocardial ultrastructure of the control group displays round nuclear myofibrils arranged in neat rows, normal muscle wire arrangement with clear cell boundaries, mitochondria of basically the same normal shape, size and arrangement; and mitochondrial cristae arranged in layers (a). The myocardial ultrastructure of the CUMS group shows myofibril breakage (b), mitochondrial disarrangement, an interrupted inner membrane and interrupted mitochondrial cristae, mitochondrial vacuoles (c, f) and nuclear membrane shrinkage and chromatin margination (d, e, f). These changes illustrate that CUMS can induce myocardial injury as well as depression-like behavior.

### 2 A Low Level of 5-HT with a High Level of GC and NE was the Neuroendocrine Pattern that Induced the Myocardial Injury

**Figure 2:**
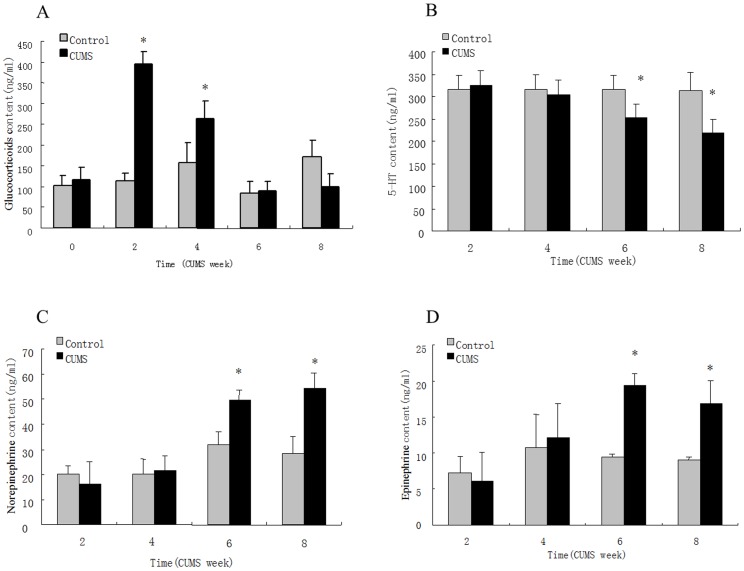
Effect of unpredictable chronic mild stress on plasma neuroendocrine transmitter in rats. The concentrations of serotonin, norepinephrine and epinephrine were measured by HPLC-ECD, and the concentration of glucocorticoid was evaluated by an I^125^–cortisol radioactivity immunoassay in both control and CUMS rats. **A.** The glucocorticoid content in plasma increased significantly after 2 and 4 weeks of CUMS but returned to normal levels after 6 and 8 weeks of CUMS. **B.** The 5-HT content in plasma increased significantly after 6 and 8 weeks of CUMS. **C.** The norepinephrine content in plasma increased significantly after 6 and 8 weeks of CUMS. **D.** The epinephrine content in plasma increased significantly after 6 and 8 weeks of CUMS. Values represent the group mean ± structural equation modeling (SEM) (n = 8 rats per group). *P<0.05 compared with control, repeated measures ANOVA followed by Tukey’s multiple comparison test.

**Figure 3:**
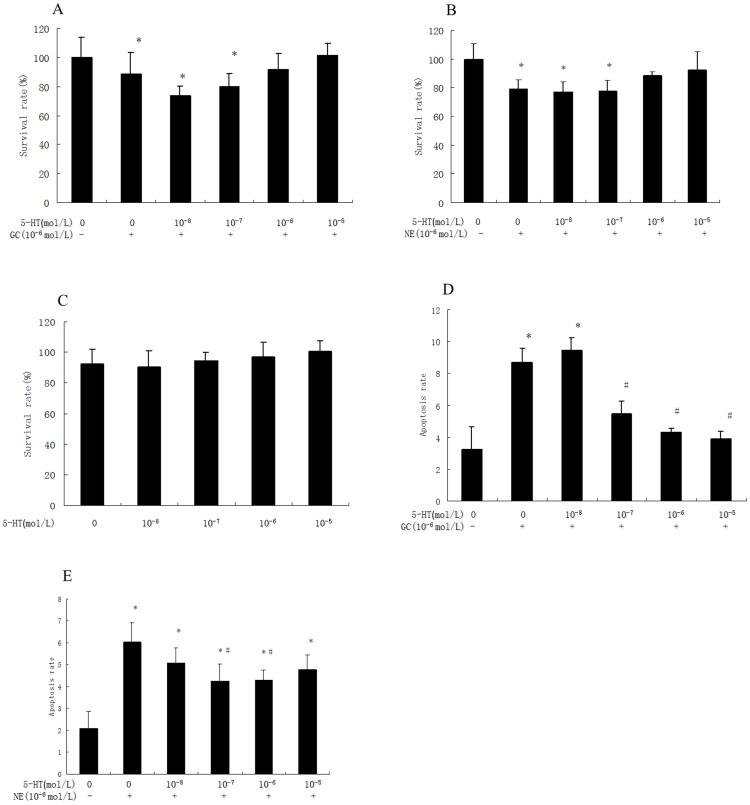
5-HT-induced cardiomyocyte injury required the presence GC or NE *in vitro*. An MTT (3-(4,5-dimethylthiazol-2-yl)-2,5-diphenyltetrazolium bromide) assay was utilized to detect cell viability. 5-HT (10^−5^, 10^−6^, 10^−7^ and 10^−8^ M) was used to treat cultured cardiomyocytes with 10^−6^ M GC or 10^−6^ M NE. **A.** The cell viability of cardiomyocytes treated with 10^−8^ M 5-HT and 10^−6^ M GC decreased significantly compared with the control. **B.** The cell viability of cardiomyocytes treated with 10^−8^ M 5-HT and 10^−6^ M NE decreased significantly compared with the control. **C.** The cell viability of cardiomyocytes treated with only 5-HT was not changed significantly compared with the control. **D.** The apoptosis rate of cardiomyocytes treated with 5-HT and 10^−6^ GC increased significantly compared with the control. **E.** The apoptosis rate of cardiomyocytes treated with 5-HT and 10^−6^ NE increased significantly compared with the control. Values represent the group mean ± structural equation modeling (SEM) (n = 8 rats per group). *P<0.05 compared with control, repeated measures ANOVA followed by Tukey’s multiple comparison test.

The synergism model of 5-HT-, GC- and NE-induced myocardial injury is not yet universally accepted. We therefore examined changes in 5-HT, GC and NE in the plasma of rats with depression and myocardial injury. The results indicated that 5-HT decreased, whereas NE, E and GC increased in CUMS rats (Fig. 2), suggesting that these factors might be associated with depression-induced myocardial injury. The effects of 5-HT, NE and GC on the survival rate of cultured cardiomyocytes were determined using an orthogonal experiment (Table 1). The results showed that 5-HT acted as the most important factor in affecting cell survival (Table 2, Fig. 3) but only if GC or NE were present (Fig. 3).

**Table 1:** The factors and the levels of the orthogonal design experiment.

		Factor		
		5-HT	GC	NE
Level	1	10^−6^ mol/L	10^−6^ mol/L	10^−6^ mol/L
	2	10^−8^ mol/L	10^−8^ mol/L	10^−7^ mol/L

Three factors and a 2-level orthogonal experiment were designed to identify the key cause of cardiomyocyte apoptosis. The factors and the levels were 5-HT (10^−^6 mol/L, 10^−8^mol/L), GC (10^−6^ mol/L, 10^−8^ mol/L) and NE (10^−6^ mol/L, 10^−7^ mol/L).

**Table 2:** Changes in the survival rate of cultured cardiomyocytes by an orthogonal experiment.

	A(5-HT)	B(GC)	A×B	C(NE)	A×C			Y(%)
1	1	1	1	1	1	1	1	96.91
2	1	1	1	2	2	2	2	90.75
3	1	2	2	1	1	2	2	97.79
4	1	2	2	2	2	1	1	108.37
5	2	1	2	1	2	1	2	81.49
6	2	1	2	2	1	2	1	87.22
7	2	2	1	1	2	2	1	70.93
8	2	2	1	2	1	1	2	89.87
I1	98.46	89.098	87.12	86.78	92.95	94.16	90.86	
II2	82.38	91.74	93.72	94.05	92.95	86.67	89.98	
R	16.08	2.65	6.60	7.273	0	7.49	0.88	
		**B1**		**B2**				
A1		93.83		103.08				
A2		84.35		80.4				
**Variation**		**SS**		***v***	**S**		***P*** ** Value**	
A(5-HT)		516.97		1	333.529		<0.01	
B(GC)		14.02		1	9.045161		>0.05	
A×B		87.19		1	56.25161		<0.05	
C(NE)		105.79		1	68.25161		<0.05	
A×C		0		1	0		>0.05	

The results of orthogonal experiments show the effects of 5-HT, GC and NE and their interaction on cell survival. 5-HT>NE >5-HT×GC>GC >5-HT×NE. 5-HT was the most important factor affecting cardiomyocyte apoptosis, followed by NE. The interaction between 5-HT and GC was the third most important factor for cardiomyocyte apoptosis. Within the dose range used, an increase of 5-HT from 10^−8^ M to 10^−6^ M promoted cell survival, but the cell survival rate decreased with increases in GC and NE.

### 3 Sertraline Hydrochloride Inhibits Depression-like Behavior and Myocardial Injury in Rats with Chronic Unpredictable Mild Stress

**Figure 4:**
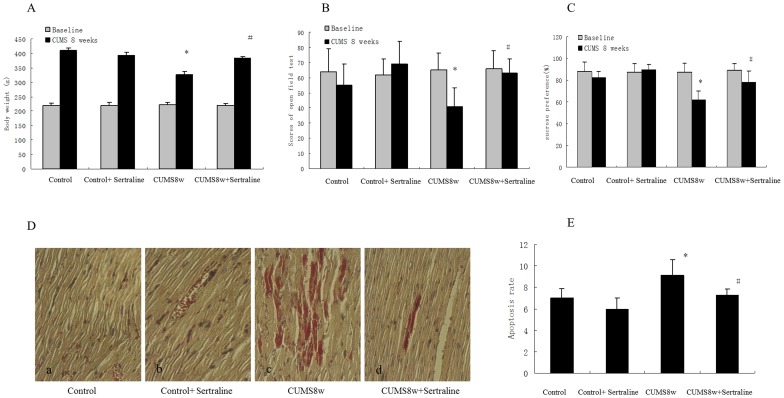
Sertraline inhibits depression-like behavior and myocardial injury in CUMS rats. Body weights, open field test scores and sucrose preference were measured as described in Figure 1. **A.** Sertraline inhibited weight loss induced by CUMS. **B.** Sertraline inhibited the decline of the open field test score induced by CUMS. **C.** Sertraline inhibited the decline of sucrose preference induced by CUMS. **D.** Sertraline inhibited the myocardium injury induced by CUMS. **E.** Sertraline inhibited the apoptosis induced by CUMS. Values represent the group mean ± structural equation modeling (SEM) (n = 8 rats per group). *P<0.05 compared with control, ^#^ *P*<0.05 compared with the 8-week CUMS group, repeated measures ANOVA followed by Tukey’s multiple comparison test.

Sertraline hydrochloride is an antidepressant of the selective serotonin reuptake inhibitor class. We found that sertraline can effectively reverse the behavioral indicators of depression and the cardiac abnormalities induced by CUMS. Sertraline can also significantly increase the body weight of CUMS rats (Fig. 4A). The total scores of the open field test are shown in Figure 4B. Sertraline significantly increased the open field test score of rats after 8 weeks of exposure to CUMS (F = 7.53, t = 3.46, df = 15, P<0.05). Sertraline also reversed the CUMS-induced decrease in sucrose preference (Fig. 4C). These results indicate that sertraline hydrochloride can effectively reverse or improve the depression-like symptoms of rats exposed to CUMS.

The rats’ myocardial tissue sections were stained with basic fuchsin to observe the effect of sertraline hydrochloride on myocardial injury. Our results showed that rats exposed to CUMS for 8 weeks had damaged cardiac histiocytes but that sertraline hydrochloride protected the rats from CUMS-induced myocardial injury (Fig. 4D).

## Discussion

Based on the etiological mechanisms of depression and heart injury, the present study investigated the comorbidity of depression and heart injury in rats with CUMS and assessed the interventional effect of sertraline. The results demonstrated that CUMS caused significantly reduced body weight and sucrose preference. CUMS rats also exhibited heart injury, increased myocardial apoptosis and cardiac morphological abnormalities. The anti-depressant sertraline effectively reversed all these CUMS-induced behavioral changes and cardiac abnormalities.

Anhedonia, which is described as a decreased ability to relate rewards with different environments [38]–[42], was inferred from decreased sucrose preference in our experiment. This behavioral change is an important indicator for the establishment of a CUMS model of depression that mimics major depression in humans and reflects responsiveness to pharmacological treatments. Rats in the current study displayed reduced sucrose preference after 4 weeks of CUMS exposure, which is consistent with previous studies [18], [28], [43]. There was no significant relationship between sucrose intake and loss of body weight in CUMS rats. The reduced sucrose preference of rats therefore reflected depression-like symptoms induced by present CUMS procedures.

The present study also demonstrates that CUMS produced the observed myocardial injury. We examined cardiac morphological structure and myocardial apoptosis. The results demonstrated that CUMS significantly damaged myocardial ultrastructure, particularly by causing mitochondrial abnormalities and then inducing myocardial apoptosis. Mitochondria are vital for maintaining the physiological function of cardiomyocytes [44]–[46]. Many stimuli can cause the mitochondrial MPTP to open [47], with a subsequent series of cytological effects that includes a loss of mitochondrial transmembrane potentials, uncoupling of the respiratory chain, leakage of mitochondrial Ca^2+^, excess generation of ROS [48], [49], release of resident mitochondrial proteins such as the apoptosis initiating factor cytochrome c and release of a second mitochondria-derived activator of caspase [50]. These effects indicate that mitochondrial damage be a key step leading to myocardial death.

Our findings confirm that CUMS can produce both depression-like symptoms and heart injury. CUMS is appropriate as a stress model to study the occurrence and mechanisms of the comorbidity of depression and heart injury [51], [52].

Plasma serotonin (5-HT) dysfunction is the most widely accepted neuroendocrine mechanism for depression [53], [54]. HPA axis dysfunction [55] and elevated levels of plasma catecholamine [56] are also hypothesized to be involved in depression. GC has complex, and often contradictory, influences on cardiovascular disease and cardiovascular risk [57]. Extensive evidence has established that NE play major role in left ventricular hypertrophy and heart failure [58]. Therapy with selective serotonin reuptake inhibitors (SSRIs) is strongly recommended to reduce cardiovascular disease-induced morbidity and mortality [59]. However, the relationship between the NE, GC and 5-HT effect on myocardial injury have not been elucidated. In this study, we find low level of 5-HT with a high level of GC and NE can induced the myocardial injury Normal blood levels of 5-HT, GC and NE have a cytoprotective effect, the neuroendocrine disorders characterized by decreased 5-HT in combination with increased GC or NE mediated depression-induced myocardial injury. We also assessed the effects of the antidepressant sertraline on depression and cardiac injury. Rats treated with sertraline 2 weeks before exposure to CUMS were found to have an increased sucrose preference after 8 weeks of CUMS exposure. However, sertraline did not completely reverse the observed reduction in body weight. This finding is further evidence of lack of an association between the reductions in sucrose intake and in body weight that are caused by CUMS.

Sertraline, a selective serotonin reuptake inhibitor (SSRI), is the most commonly prescribed therapy for maternal depression. Some studies have linked SSRI exposure with myocardial protection [60]. On the other hand, it was investigated that sertraline is a potent blocker of cardiac K^+^ channels and the patients taking sertraline especially with risks of long QT syndrome should be cautiously monitored for clinical signs of cardiac arrhythmia [61]. The present results of myocardial apoptosis screening also show that sertraline hydrochloride confers significant protection against myocardial cell injury (Fig. 4D). This activity suggests that sertraline hydrochloride can effectively protect CUMS rats from myocardial pathological injury by reducing cardiomyocyte apoptosis. The decrease in the 5-HT level might be the most important cause of myocardial injury in CUMS rats, and increased levels of GC and NE may be contributive. Therefore, we presumed that sertraline might be an ideal drug for the treatment of comorbid depression and cardiovascular diseases in humans. The mechanism of sertraline treatment for both disorders may include direct and indirect effects. Vasodilatory, antiplatelet and endothelium- protective effects of sertraline [62], [63] may contribute to the direct effect. The indirect effect may result from the use of sertraline to treat depression, and recovery from depression might contribute to an amelioration of heart injury.

In conclusion, CUMS is an effective animal model for studying the comorbidity of depression and heart injury. The present research indicates that depressive behavior can cause myocardial injury. The neuroendocrine disorders characterized by decreased 5-HT in combination with increased GC or NE is the physiology mechanism of depression-induced myocardial injury. Sertraline, which had previously been shown to be an effective anti-depressant, is now confirmed to be a suitable treatment for human patients suffering simultaneously from depression and cardiovascular disease.
